# Increased zinc levels facilitate phenotypic detection of ceftazidime-avibactam resistance in metallo-β-lactamase-producing Gram-negative bacteria

**DOI:** 10.3389/fmicb.2022.977330

**Published:** 2022-11-22

**Authors:** Michaela Simon, Roman G. Gerlach, Yvonne Pfeifer, Niels Pfennigwerth, Sören G. Gatermann, Agnes Schröder, Andreas Hiergeist, Axel Hamprecht, Tamara Rügamer, André Gessner, Jonathan Jantsch

**Affiliations:** ^1^Institute of Clinical Microbiology and Hygiene, University Hospital of Regensburg, Regensburg, Germany; ^2^Institute for Medical Microbiology, Immunology, and Hygiene, University Hospital Cologne and Faculty of Medicine, University of Cologne, Cologne, Germany; ^3^Mikrobiologisches Institut-Klinische Mikrobiologie, Immunologie und Hygiene, Universitätsklinikum Erlangen and Friedrich-Alexander-Universität (FAU) Erlangen- Nürnberg, Erlangen, Germany; ^4^FG13 Nosocomial Pathogens and Antibiotic Resistance, Robert Koch Institute, Wernigerode, Germany; ^5^Department of Medical Microbiology, German National Reference Centre for Multidrug-Resistant Gram-negative Bacteria, Ruhr-University Bochum, Bochum, Germany; ^6^Department of Orthodontics, University Hospital Regensburg, Regensburg, Germany; ^7^Department of Medical Microbiology and Virology, Carl von Ossietzky University Oldenburg, Oldenburg, Germany; ^8^Institute for Medical Microbiology and Virology, Oldenburg, Germany; ^9^German Centre for Infection Research, Partner Site Bonn-Cologne, Cologne, Germany

**Keywords:** Enterobacterales, *Pseudomonas aeruginosa*, metallo-β-lactamases, ceftazidime-avibactam, zinc, antimicrobial susceptibility testing, Phoenix, Micronaut

## Abstract

Ceftazidime-avibactam is one of the last resort antimicrobial agents for the treatment of carbapenem-resistant, Gram-negative bacteria. Metallo-β-lactamase-producing bacteria are considered to be ceftazidime-avibactam resistant. Here, we evaluated a semi-automated antimicrobial susceptibility testing system regarding its capability to detect phenotypic ceftazidime-avibactam resistance in 176 carbapenem-resistant, metallo-β-lactamase-producing Enterobacterales and *Pseudomonas aeruginosa* isolates. Nine clinical isolates displayed ceftazidime-avibactam susceptibility in the semi-automated system and six of these isolates were susceptible by broth microdilution, too. In all nine isolates, metallo-β-lactamase-mediated hydrolytic activity was demonstrated with the EDTA-modified carbapenemase inactivation method. As zinc is known to be an important co-factor for metallo-β-lactamase activity, test media of the semi-automated antimicrobial susceptibility testing system and broth microdilution were supplemented with zinc. Thereby, the detection of phenotypic resistance was improved in the semi-automated system and in broth microdilution. Currently, ceftazidime-avibactam is not approved as treatment option for infections by metallo-β-lactamase-producing, Gram-negative bacteria. In infections caused by carbapenem-resistant Gram-negatives, we therefore recommend to rule out the presence of metallo-β-lactamases with additional methods before initiating ceftazidime-avibactam treatment.

## Introduction

Multidrug-resistant Gram-negative bacteria (MDR-GN) are spreading globally (Laxminarayan et al., 2013). Carbapenem resistance in MDR-GN is mediated by carbapenemases or combined resistance mechanisms; i.e., altered membrane permeability plus production of extended-spectrum β-lactamase (ESBL) or AmpC β-lactamase (AmpC; Palzkill, 2013; EUCAST, 2017). The novel β-lactam/β-lactamase inhibitor combination ceftazidime-avibactam targets many β-lactamases including ESBL, AmpC, Ambler Class A, and OXA-48-type carbapenemases (Livermore et al., 2018). However, avibactam does not inhibit Ambler Class B carbapenemases, also known as metallo-β-lactamases (MBLs; Palzkill, 2013; Rodriguez-Bano et al., 2018). Common MBLs include IMP-type (Imipenemase Metallo-β-lactamase), NDM-type (New Delhi Metallo-β-lactamase) and VIM-type (Verona Integron Metallo-β-lactamase) carbapenemases. MBLs occur worldwide in carbapenemase-producing Enterobacterales and especially *Pseudomonas aeruginosa* isolates (Boyd et al., 2020) and convey ceftazidime-avibactam resistance (Livermore et al., 2011).

In routine microbiological laboratories, detection and specification of carbapenemases are usually subsequent steps after phenotypic antimicrobial susceptibility testing (AST; Simon et al., 2019). Therefore, it is crucial that phenotypic AST systems can identify ceftazidime-avibactam resistance of MBL producers. Here, we investigated the capability of a semi-automated AST system to detect ceftazidime-avibactam resistance in 176 MBL-producing MDR-GN.

## Materials and methods

### Study isolates

The isolate collection of MBL-positive MDR-GN included 42 *Pseudomonas aeruginosa* and 134 Enterobacterales isolates (57 *Klebsiella pneumoniae*, 38 *Enterobacter cloacae* complex, 15 *Escherichia coli*, 13 *Klebsiella oxytoca* complex, 4 *Citrobacter freundii* complex, 4 *Proteus mirabilis*, 1 *Morganella morganii*, 1 *Providencia stuartii*, 1 *Serratia marcescens*). Thirty-five isolates derived from clinical specimens were processed at the Institute of Clinical Microbiology and Hygiene, Regensburg, Germany. The Robert Koch Institute, Wernigerode, Germany, provided 140 clinical isolates and the German National Reference Centre for Multidrug-Resistant Gram-Negative Bacteria one clinical isolate. The test isolates were stored at −80°C and grown overnight on Columbia agar plates +5% sheep blood (Oxoid, Cambridge, United Kingdom) at a temperature of 35°C ± 1°C in ambient air.

### Establishing ceftazidime-avibactam resistance

All 176 isolates harbored genes encoding MBLs (IMP-type: *n* = 3, NDM-type: *n* = 61, VIM-type: *n* = 112); therefore, phenotypic ceftazidime-avibactam resistance corresponding to a minimal inhibitory concentration (MIC) of >8/4 mg/L (EUCAST, 2022) was expected in all isolates when tested with the semi-automated AST system. MBL gene presence was determined by PCR and Sanger Sequencing (Supplementary Table 1) or using the real-time PCR system Xpert Carba-R, version 2 (Cepheid, Sunnyvale, United States).

### Phoenix M50 semi-automated AST system

Semi-automated testing (SAT) was performed using the Phoenix M50 device (Becton Dickinson, Heidelberg, Germany). The Phoenix AP instrument was used according to the manufacturer’s recommendation to inoculate NMIC-502 AST panels with BD Phoenix AST broth (SAT medium). The NMIC-502 panels include the CPO (carbapenemase-producing organism) Detect assay (Simon et al., 2019). This growth-based assay is an integral part of the panel and works with various β-lactamase inhibitors in order to identify carbapenemase activity. Every NMIC-502 panel test run provides a CPO Detect result. In this way, every test run is automatically checked for carbapenemase activity. In addition to the positive CPO Detect assay, test strains with ceftazidime-avibactam susceptibility were re-tested at least once with the Xpert Carba-R system in order to confirm the presence of MBL genes. For this, the bacterial suspensions prepared in Phoenix ID broth for the inoculation of NMIC-502 panels were stored at −20°C. The minimum and maximum measurable ceftazidime-avibactam MICs are ≤0.25/4 and > 8/4 mg/L, respectively. The concentration of avibactam in the NMIC-502 AST panels is fixed at 4 mg/L.

All isolates were tested in two independent test runs. If one of these test runs resulted in ceftazidime-avibactam susceptibility, two further independent test runs were performed. The modal categorical test result (susceptible or resistant) was considered the final result.

MBL-positive isolates with an MIC in the susceptible range of ceftazidime-avibactam (i.e., MIC ≤8/4 mg/L) were re-evaluated in the semi-automated AST (SAT) system in triplicates on 5 days using zinc-supplemented and non-supplemented SAT medium in parallel. For this, a 35 mM ZnSO_4_ 7 H_2_O solution (Merck, Darmstadt, Germany) was prepared with distilled, sterile water. The zinc content of the SAT medium was elevated with 0.0682, 0.1137, 0.1365, 0.1706, or 0.2274 mM zinc (corresponding to 4.46, 7.44, 8.92, 11.15, and 14.87 mg/L), respectively.

Furthermore, two Enterobacterales and two *P. aeruginosa* strains harboring NDM-1, VIM-1, VIM-2, and IMP-1, respectively, with ceftazidime-avibactam resistance in SAT were re-evaluated with and without the chelating agent ethylenediaminetetraacetic acid (EDTA)-supplemented test medium. EDTA (Merck, Darmstadt, Germany) was added in a final concentration of 300 mg/L (Asempa et al., 2020). For the two *P. aeruginosa* strains, EDTA was reduced to 50 and 150 mg/L, respectively, because these EDTA concentrations did not interfere with bacterial growth in the SAT system. Broth microdilution (BMD) with and without EDTA supplementation was performed in parallel.

### Broth microdilution

BMD was performed using the Micronaut-S system (Merlin Diagnostika GmbH, Bornheim, Germany) according to the manufacturer’s recommendation. In brief, bacterial suspensions corresponding to a turbidity standard of 0.5 according to McFarland were prepared and 50 μL were transferred to 11.5 mL of Micronaut-S cation-adjusted Mueller–Hinton broth (CAMHB; BMD medium). 100 μL per well were inoculated into a 96-well plate containing freeze-dried antibiotics including ceftazidime-avibactam (range 1–8/4 mg/L). After 18–24 h of incubation at 35°C ± 1°C MICs were read with the Tecan Sunrise photometer (Sifin Diagnostics GmbH, Berlin, Germany) using the MCN6 software (version 6.00, release 112, DEMOS Computer GmbH, Merlin Diagnostika).

MBL producers with susceptible results in the SAT system were subjected to BMD in at least three independent test runs. If the modal MIC was ≤8/4 mg/L, MBL genes were confirmed with the Xpert Carba-R system and BMD was repeated with zinc-supplemented BMD medium.

Four strains showing ceftazidime-avibactam resistance in the SAT system were re-tested with and without EDTA-supplemented test medium. As described previously, EDTA was added in 300, 150, and 50 mg/L, respectively, and SAT was performed in parallel.

### Confirmation of MBL-mediated hydrolysis

MBL-positive isolates with susceptible ceftazidime-avibactam results in SAT were examined with the modified carbapenemase inactivation method (mCIM) and the EDTA-modified CIM (eCIM) in order to confirm expression of MBLs and their enzymatic activity. mCIM and eCIM were performed according to the Clinical and Laboratory Standards Institute (CLSI) (2019) and Gill et al. (2020b). Briefly, 10 μg meropenem disks (Becton Dickinson, Heidelberg, Germany) were incubated in a suspension of the test isolates in tryptic soy broth (Merck, Darmstadt, Germany) with and without EDTA in a final concentration of 5 mM. After 4 h of incubation, the disks were placed on Mueller–Hinton E agar plates (bioMérieux, Marcy l’Etoile, France), which had been inoculated with a McFarland 0.5 suspension of the meropenem-susceptible strain *E. coli* ATCC 25922 by streaking in three directions. After 18 h of incubation, the inhibition zone diameters were measured and interpreted according to specifications provided by Gill et al. and CLSI. A positive test result indicates MBL-mediated hydrolysis of meropenem.

### Interpretation of minimum inhibitory concentrations

MIC values were categorized as susceptible, standard exposure (≤8/4 mg/L), or resistant (>8/4 mg/L) according to EUCAST breakpoints (EUCAST, 2022).

### Quality control

Quality control of AST was performed with *P. aeruginosa* ATCC 27853 and *K. pneumoniae* ATCC 700603. In all test runs (with and without zinc supplementation), MIC values of ceftazidime-avibactam were within the acceptable ranges.

### Evaluation of the zinc concentration in the SAT and BMD medium

After dilution in 0.5% caesium chloride (CsCl; 289329, Sigma-Aldrich, Schnelldorf, Germany), zinc concentration was measured by atomic absorption spectrometry (iCE 3500, Thermo Fisher Scientific). Zinc-atomic absorption spectrometry (AAS) standard solution (2383.1) was purchased from Carl Roth (Karlsruhe, Germany). Samples were pre-diluted by a factor of 3 with 0.5% CsCl. Measurements were performed in triplicates. The coefficient of variation was between 0.1% and 2.0%. The range of the standard curve was 0.0031 to 0.0184 mM (without pre-dilution factor).

### Quantitative bacterial culture

Tubes with 4.5 mL SAT medium were supplemented with zinc (0.0682, 0.1137, 0.1365, 0.1706, and 0.2274 mM) and inoculated with 10 μL of a bacterial suspension with a density of a McFarland 0.5 turbidity standard. After overnight incubation at 35°C ± 1°C in ambient air, the liquid culture was diluted 1:1,000,000 with saline and 100 μL were plated on Mueller–Hinton 2 agar plates (bioMérieux, Marcy l’Etoile, France). After overnight incubation at 35°C ± 1°C in ambient air, colony-forming units were counted and the bacterial concentration was calculated.

### Whole-genome sequencing

Genomic DNA was isolated from 2 mL overnight culture in LB (Luria-Bertani) broth using the GenElute Bacterial Genomic DNA kit (Sigma-Aldrich) according to the manufacturer’s recommendations. One nanogram DNA was fragmented by tagmentation using the Nextera XT kit (Illumina, Berlin, Germany). Libraries were dual-indexed by PCR and subjected to 2 × 150 bp paired-end sequencing on a NextSeq 550 instrument (Illumina). Adapter sequences were removed, and sequence data were filtered based on quality with BBDuk from BBMap v38.79.^1^ SKESA v2.3.0 (Souvorov et al., 2018) was applied for *de novo* assembly from filtered short reads. DNA of *E. coli* isolates 371.12 and 700.18, *M. morganii* 64.08, *P. mirabilis* 52.15, and *P. aeruginosa* 613.16 was extracted from 5 mL overnight culture in LB broth using the High Pure PCR template preparation kit (Roche Molecular Systems, Pleasanton, United States). Short sequencing reads were obtained from IonTorrent-based semiconductor sequencing. Here, a total of 1 μg DNA was fragmented to an average of 350 bp by focussed-ultrasonication with the Covaris ME220 instrument (Covaris, Brighton, United Kingdom). The Ion Plus Fragment Library Kit (Thermo Fisher Scientific, Waltham, United States) was used for end repair of DNA fragments and subsequent ligation of Ion Xpress™ barcodes and sequencing adapters. Automated preparation of sequencing libraries was conducted on an IonChef™ instrument followed by high-throughput sequencing on an IonTorrent™ Genestudio S5 Plus sequencer (Thermo Fisher Scientific) resulting in a mean genomic coverage above 100-fold. Long reads were obtained by nanopore sequencing on a MinION instrument (Oxford Nanopore Technologies, Oxford, United Kingdom) starting from 3 μg of DNA. The Ligation Sequencing Kit (SQK-LSK109) was used for preparation of sequencing libraries, which included removal of DNA fragments smaller than 3 kb. Sequencing was performed on a R9.4.1 flow cell until a minimum of 20-fold mean genomic coverage was reached. Hybrid assembly of long and short reads was carried out with Unicycler v0.4.8 (Wick et al., 2017). Sequence data are available through the National Center for Biotechnology Information (NCBI) Sequence Read Archive (SRA) under BioProjects PRJNA869506 and PRJNA871076. The number of contigs after assembly and genome accession numbers are shown in Supplementary Table 2.

### Phylogenetic analysis

Whole-genome single-nucleotide polymorphism (SNP) alignments based on the contigs were done for each species with PhaME v1.0.2 (Shakya et al., 2020) using *E. coli* isolates K-12 substr. MG1655 (GenBank: NC_000913.3), O157:H7 str. Sakai (GenBank: BA000007.3), ATCC 11775 (GenBank: CP033092.2) or *P. aeruginosa* isolate PAO1 (GenBank: NC_002516.2) as references, respectively. Approximately maximum-likelihood trees were calculated for the isolates with FastTree 2 (Price et al., 2010) and rooted at midpoint.

### Detection of AMR genes

Genes conferring antimicrobial resistance were identified in the assembled genomes with ABRicate v1.0.1 (Seemann T.)^2^ using the ResFinder database (3,139 entries after filtering, accessed 2021-12-03) (Bortolaia et al., 2020). The percentages of coverage and identity of β-lactamase genes to reference sequences were extracted from the ABRicate output. Genes with less than 100% coverage and/or identity were further subjected to Basic Local Alignment Search Tool (BLAST; Altschul et al., 1990) against the “nr” nucleotide database (accessed 2022-02-08) to identify reference sequences not present in ResFinder database.

### Statistical analysis

Very major error (VME) rates were determined following ISO 20776-2 (2007) using the presence of MBL genes as a reference for ceftazidime-avibactam resistance:


$$VME=\frac{\begin{array}{l} numberof\;MBL-positiveisolates\\ classified\;as\;susceptible\;by\;Phoenix*100 \end{array}}{numberof\;all\;tested\;MBL-positiveisolates}$$

Continuous variables are given as mean ± standard deviation (SD). Statistical analysis was performed with the Kruskal–Wallis test for continuous variables using IBM SPSS (version 28.0, IBM, Armonk, United States).

## Results

### Evaluation of phenotypic ceftazidime-avibactam resistance in MBL-harboring isolates

The isolates of our collection comprising 176 MBL-positive MDR-GN isolates were subjected to SAT. All test isolates displayed phenotypic resistance toward at least one carbapenem (meropenem, imipenem, and for Enterobacterales additionally ertapenem) when tested with the SAT system (Supplementary Table 3). For Enterobacterales, carbapenem MIC_50_ values were identical to MIC_90_ values (>1 mg/L for ertapenem, range ≤0.25 to >1 mg/L; >8 mg/L for imipenem, range 0.5 to >8 mg/L; >8 mg/L for meropenem, range 0.5 to >8 mg/L). For *P. aeruginosa*, carbapenem MIC_50_ and MIC_90_ values were also identical (>8 mg/L for imipenem, all values >8 mg/L; >8 mg/L for meropenem, range 4 to >8 mg/L).

The SAT system detected ceftazidime-avibactam resistance in 167 isolates (Table 1). With the presence of MBL genes as an indicator for resistance, this corresponds to an overall VME rate of 5.1% (9/176). The VME rate for Enterobacterales was 3.7% (5/134). False-susceptible Enterobacterales were one *E. cloacae* complex (*E. hormaechei* subsp. *hoffmannii*) with VIM-1 (strain no. KR29), two *E. coli* isolates harboring NDM-1 (strain no. 2.10) and NDM-5 (strain no. 700.18), respectively, one *M. morganii* with VIM-4 (strain no. 64.08) and one *P. mirabilis* with VIM-1 (strain no. 52.15). Four VIM-2-harboring *P. aeruginosa* isolates (strains no. AV6, AV7, 613.16, WE10) were classified as ceftazidime-avibactam susceptible resulting in a VME rate of 9.5% (4/42), again when MBL genes are considered to indicate resistance (Table 1).

**Table 1:** VME rates in ceftazidime-avibactam semi-automated susceptibility testing of MBL-producing MDR-GN with MBL genes as indicator for phenotypic resistance.

Organisms (*n*)	VME % (*n*)
Overall (176)	5.1 (9)
Enterobacterales (134)	3.7 (5)
NDM-type (61)	3.3 (2)
VIM-type (73)	4.1 (3)
*Pseudomonas aeruginosa* (42)	9.5 (4)
IMP-type (3)	0
VIM-type (39)	10.3 (4)

VME, very major error; MDR-GN, multidrug-resistant Gram-negative bacteria; MBL, metallo-β-lactamase; *n*, number of isolates; NDM, New Delhi Metallo-β-lactamase; VIM, Verona Integron Metallo-β-lactamase; IMP, Imipenemase Metallo-β-lactamase.

**Figure 1:**
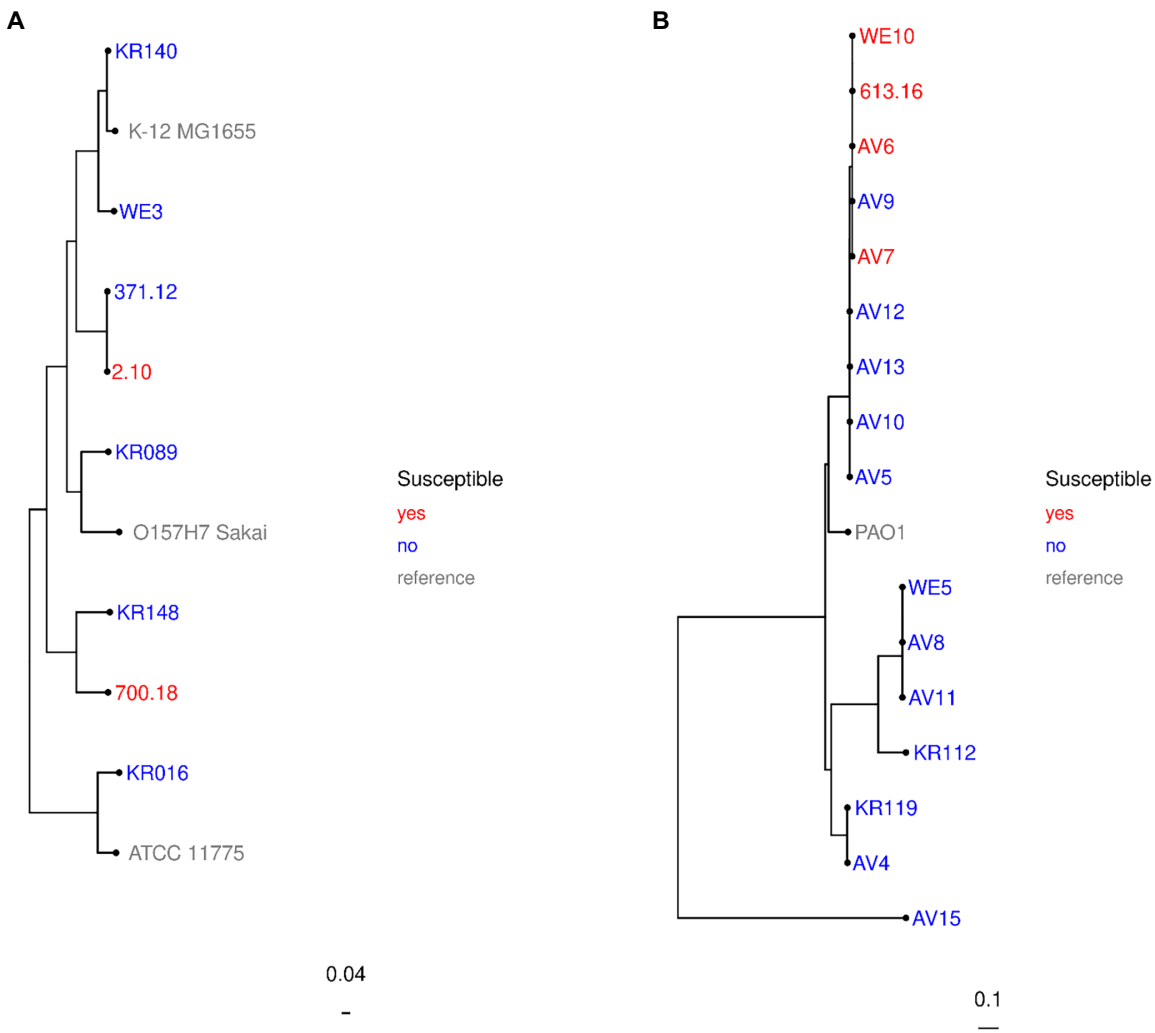
Phylogenetic relationship of investigated strains: *Escherichia coli* **(A)** and *Pseudomonas aeruginosa* **(B)**. Whole-genome single-nucleotide polymorphism (SNP) alignments based on the contigs were done for each species with PhaME v1.0.2 (Shakya et al., 2020). Approximately maximum-likelihood trees were calculated for the isolates with FastTree 2 (Price et al., 2010) and rooted at midpoint. Created by R. Gerlach for this study.

For further molecular characterization of the β-lactamase content and the genetic relationship, we performed whole genome sequencing (WGS) of these nine isolates. The subtypes of β-lactamase genes are given in Table 2. Analysis of coverage and identity to reference sequences revealed 100% concordance for all MBL genes except bla_NDM-1_ of *E. coli* strain no. 2.10 and bla_VIM-4_ of *M. morganii* strain no. 64.08. While bla_VIM-4_ was 5′-truncated by 63 bp at a contig start, for bla_NDM-1_ of isolate no. 2.10 a single base pair deletion (ΔT207) was found. However, this deletion was not confirmed by Sanger sequencing (data not shown) and can likely be attributed to an Illumina sequencing error. Whole-genome SNP-based phylogenetic analysis determined no close relationship between the two *E. coli* isolates (Figure 1A). For *P. aeruginosa*, phylogenetic analysis revealed genomic relationship of these isolates (Figure 1B). Three isolates were isolated from clinical samples in Regensburg. The fourth isolate was provided by the Robert Koch Institute and was recovered from another site in Southern Germany, suggesting that at least regional significance of the clone is possible. Further MBL-producing isolates of this cluster (strain no. AV5, AV9, AV10, AV12, AV13; Figure 1B) had been correctly classified as resistant in the SAT system. Based on the epidemiological data, it cannot be ruled out that the clone is more widespread than just at a single site.

**Table 2:** Detected β-lactamase genes of nine isolates with false-susceptible ceftazidime-avibactam results in the semi-automated AST system.

Strain no.	Organism	β-lactamases
KR29	*E. cloacae* complex	**VIM-1 (100/100)**, ACT-14, SHV-12
700.18	*E. coli*	**NDM-5 (100/100)**, CTX-M-15, TEM-1B
2.10	*E. coli*	**NDM-1 (99.88/99.88)**, CTX-M-15, OXA-1, OXA-2, TEM-1B
64.08	*M. morganii*	**VIM-4 (92.3/100)**, DHA-4
52.15	*P. mirabilis*	**VIM-1 (100/100)**, CMY-99, SHV-12, TEM-1A
613.16	*P. aeruginosa*	**VIM-2 (100/100)**, OXA-395, PDC-44
AV6	*P. aeruginosa*	**VIM-2 (100/100)**, OXA-395, PDC-44
AV7	*P. aeruginosa*	**VIM-2 (100/100)**, OXA-395
WE10	*P. aeruginosa*	**VIM-2 (100/100)**, OXA-395, PDC-44

Metallo-β-lactamases are given in bold type and percentages of coverage and identity to reference sequences are given in brackets (coverage %/identity %).

For further phenotypic characterization of the nine strains with ceftazidime-avibactam susceptibility in the SAT system, they were subjected to BMD (Table 3). However, only three strains (*E. cloacae* complex strain no. KR29 with VIM-1, *E. coli* strain no. 700.18 with NDM-5, and *E. coli* strain no. 2.10 with NDM-1) exhibited ceftazidime-avibactam resistance by BMD. The remaining six strains were ceftazidime-avibactam susceptible, although loss of MBL genes was excluded with real-time PCR. This could indicate lacking enzyme expression or expression of non-functional carbapenemases. However, the inhibitor-based CPO Detect assay, which is included in the test panels of the SAT system and detects carbapenemase-mediated hydrolysis, categorized these six strains as carbapenemase producers. In addition, the eCIM was performed, which is recommended by CLSI and detects hydrolytic activity of MBLs (Clinical and Laboratory Standards Institute (CLSI), 2019). All six strains with ceftazidime-avibactam susceptibility by BMD were positive by eCIM (Table 3). The three strains with ceftazidime-avibactam resistance by BMD and susceptibility by SAT were eCIM-positive, too. This suggests that all isolates with ceftazidime-avibactam susceptibility in one or both phenotypic AST systems actually express functional MBLs.

**Table 3:** eCIM and phenotypic ceftazidime-avibactam antimicrobial susceptibility testing with and without zinc addition of nine MBL-producing MDR-GN.

				Semi-automated AST system		Broth microdilution	
Strain no.	Organism	MBL	eCIM	Zinc addition (mM)	Phenotypic resistance detected	Zinc addition (mM)	Phenotypic resistance detected
KR29	*E. cloacae* complex	VIM-1	+	0	No	0	Yes
				0.0682	Yes		
700.18	*E. coli*	NDM-5	+	0	No	0	Yes
				0.0682–0.2274	No		
2.10	*E. coli*	NDM-1	+	0	No	0	Yes
				0.0682	Yes		
64.08	*M. morganii*	VIM-4	+	0	No	0	No
				0.1137	Yes	0.1137	Yes
52.15	*P. mirabilis*	VIM-1	+	0	No	0	No
				0.1706	Yes	0.0682–0.2274	No^*^
						0.1706 and 0.2274	Yes^**^
613.16	*P. aeruginosa*	VIM-2	+	0	No	0	No
				0.0682	Yes	0.0682	Yes
AV6	*P. aeruginosa*	VIM-2	+	0	No	0	No
				0.0682	Yes	0.0682	Yes
AV7	*P. aeruginosa*	VIM-2	+	0	No	0	No
				0.0682	Yes	0.0682	Yes
WE10	*P. aeruginosa*	VIM-2	+	0	No	0	No
				0.1706	Yes	0.0682	Yes

eCIM, EDTA-modified carbapenemase inactivation method; MDR-GN, multi-drug resistant Gram-negative bacteria; AST, antimicrobial susceptibility testing; no., number; MBL, metallo-β-lactamase; VIM, Verona Integron Metallo-β-lactamase; NDM, New Delhi Metallo-β-lactamase; 0.0682 mM = 4.46 mg/L; 0.2274 mM = 14.87 mg/L; 0.1137 mM = 7.44 mg/L; 0.1706 mM = 11.15 mg/L.

* With BMD medium.

** With SAT medium.

### Re-evaluation of ceftazidime-avibactam susceptible, MBL-producing isolates using zinc-supplemented test media

As eCIM revealed functional MBLs, which are considered to mediate ceftazidime-avibactam resistance, susceptibility by phenotypic AST, especially BMD, was unexpected. We hypothesized that the zinc content of AST media might be a crucial factor for MBL-mediated resistance (Livermore and Woodford, 2000). Therefore, the isolates with ceftazidime-avibactam susceptibility in AST at standard zinc concentrations were subjected to AST using zinc-supplemented test media. In the SAT system, addition of zinc to the SAT medium facilitated correct identification of ceftazidime-avibactam resistance in eight of the nine susceptible isolates (Table 3). In one NDM-5-positive *E. coli* isolate (strain no. 700.18), reliable detection of phenotypic ceftazidime-avibactam resistance was not possible even after adding zinc. Since zinc might impair the multiplication of this isolate, which might ultimately result in partially restricted growth in the SAT system and mimic phenotypic susceptibility, we performed bacterial growth assays. The growth assays excluded this possibility and revealed that increase of zinc in the range 0.0682–0.2274 mM did not affect bacterial proliferation (Supplementary Table 4).

Since strain no. 700.18 was ceftazidime-avibactam resistant by BMD without zinc supplementation of the BMD medium baseline concentrations of the BMD and SAT media may be significantly different. Both manufacturers do not disclose the exact zinc content. Therefore, the zinc contents of eight batches SAT medium and four batches BMD medium were determined by atomic absorption spectrometry. Mean zinc concentrations in the SAT and BMD medium were comparable (0.019 ± 0.002 mM, corresponding to 1.21 ± 0.12 mg/L, and 0.017 ± 0.001 mM, corresponding to 1.09 ± 0.07 mg/L, for SAT and BMD medium, respectively). Thus, for *E. coli* no. 700.18 with functional NDM-5, standard zinc concentrations seem to be sufficient for MBL activity in BMD, but in the SAT system, additional factors seem to play a role. Nevertheless, our findings demonstrate that in the majority of the ceftazidime-avibactam susceptible isolates increased zinc levels allowed for correct identification of ceftazidime-avibactam resistance in the SAT system (Table 3).

Also in BMD, zinc supplementation of the BMD medium resulted in enhanced detection of ceftazidime-avibactam resistance in five of six strains (Table 3), with the exception of *P. mirabilis* (strain no. 52.15, VIM-1), which gave inconsistent results in zinc-supplemented BMD with detection rates of ≤50% depending on how much zinc had been used. However, detection of resistance had been reliable in the SAT system with addition of 0.1706 mM zinc. Therefore, *P. mirabilis* no. 52.15 was subjected to BMD again, but this time using the BMD medium (with and without zinc) and the SAT medium (with and without zinc) in parallel. This was to examine, if the SAT medium includes additional components, which can, together with elevated zinc levels, promote the phenotypic detection of ceftazidime-avibactam resistance, at least in this individual strain. BMD was performed in three independent test runs using standard and zinc-supplemented (0.0682–0.2274 mM) SAT and BMD medium in parallel. BMD with zinc-supplemented SAT medium at 0.1706 or 0.2247 mM gave uniformly resistant results (Table 3). This suggests that ceftazidime-avibactam AST of *P. mirabilis* no. 52.15 is enhanced by the combination of elevated zinc levels with further components in the SAT medium.

In order to support the hypothesis that zinc plays a key role in the detection of phenotypic resistance, we tested strains classified as ceftazidime-avibactam resistant by SAT with un-modified test media again and applied EDTA as a chelator for functional zinc depletion of the test media (Supplementary Table 5). For *E. coli* strain no. 162.10 (VIM-1), *K. pneumoniae* strain no. 39.11 (NDM-1), *P. aeruginosa* strain no. 82.10 (IMP-1), and *P. aeruginosa* strain no. 52.18–1 (VIM-2), SAT and BMD were performed in parallel with and without EDTA supplementation of the test media (*n* = 4). Ceftazidime-avibactam and meropenem were classified resistant in all test runs without EDTA addition except for meropenem in BMD of *E. coli* strain no. 162.10. Test runs with zinc-depleted media resulted in a reduction of the ceftazidime-avibactam and meropenem MICs by a factor of 8–64. This was also true for BMD of meropenem and strain no. 162.10, although MICs without EDTA were in the category S and I, respectively. EDTA addition reduced the meropenem MICs of strain no. 162.10 from 2–4 mg/L (without EDTA) to ≤0.125 mg/L (with EDTA). Altogether, EDTA resulted in significant declines of the meropenem and ceftazidime-avibactam MICs of all four strains by both AST methods.

Overall, our findings indicate that in MBL producers, phenotypic detection of ceftazidime-avibactam resistance can be challenging and that zinc supplementation of AST media can facilitate detection of ceftazidime-avibactam resistance with phenotypic AST methods.

## Discussion

Since treatment options for infections caused by carbapenem-resistant MDR-GN are limited, ceftazidime-avibactam is a key agent in the therapy of infections caused by carbapenemase-producing MDR-GN. However, MBL-producing MDR-GN are considered to be ceftazidime-avibactam resistant (Rodriguez-Bano et al., 2018). Therefore, we focused on MDR-GN harboring IMP-, NDM-, or VIM-type carbapenemases and investigated the capability of a commercial SAT system to detect phenotypic ceftazidime-avibactam resistance in MBL producers.

When MBL genes were used to indicate ceftazidime-avibactam resistance, the overall VME rate was 5.1% (9/176). For Enterobacterales and *P. aeruginosa,* the VME rates were 3.7% (5/134) and 9.5% (4/42), respectively (Table 1). This exceeds the maximum acceptable VME rate of 3% stated in ISO standard 20776–2 (ISO 20776-2, 2007). The nine MBL producers categorized as susceptible in the SAT system were additionally subjected to BMD for confirmation of phenotypic ceftazidime-avibactam resistance (Table 3). Although presence of MBL genes was, again, confirmed with real-time PCR, only three of nine strains (Table 3) displayed phenotypic resistance in BMD, whereas two Enterobacterales and four *P. aeruginosa* strains were ceftazidime-avibactam susceptible by BMD as well. Phylogenetic analysis determined that these four *P. aeruginosa* strains were genetically related, possibly suggesting that the VME rate might be attributable to a local clonal outbreak. However, a recent German multi-center study reported nine of 122 MBL-producing *P. aeruginosa* (7.4%) to be ceftazidime-avibactam susceptible and meropenem resistant in BMD (Manzke et al., 2022). Therefore, VIM-harboring *P. aeruginosa* with ceftazidime-avibactam susceptibility by BMD can likely be found throughout Germany.

The absence of phenotypic resistance in presence of β-lactamase genes can be caused by impaired gene expression or expression of non-functional enzymes. Therefore, eCIM was performed, which confirmed the hydrolytic activity of MBLs in all nine strains (Table 3). Thus, inactive MBLs were not likely to be the cause of ceftazidime-avibactam susceptibility. Another explanation for phenotypic susceptibility despite presence of MBL genes could be the zinc dependence of MBLs. Zinc enables MBLs to bind and hydrolyze β-lactams and is also required for the structural stability of MBLs in the periplasmic space (Bahr et al., 2021, 2022). Therefore, we assumed that standard zinc contents of the AST media might be too low for stable and functional enzymes in a subset of MBL-producing MDR-GN. On the other hand, the majority of strains in our collection appeared to have zinc requirements that were covered by the SAT medium with standard zinc content, because they were correctly classified as resistant. We hypothesized that providing excessive zinc for the false-susceptible strains by supplementation of the test media might enhance the hydrolytic capability of MBLs, so that the phenotypic AST systems might be able to identify ceftazidime-avibactam resistance. Previous studies support this assumption, which showed that increasing the zinc content improved the sensitivity of diagnostic assays targeting the detection of MBLs (Dortet et al., 2014; Hamprecht et al., 2018; Saleh et al., 2018; Baeza et al., 2019).

The SAT and BMD media have comparable zinc contents of 0.019 and 0.017 mM, respectively. These values are within the concentration range (0.299–7.8 mg/L) of other solid and liquid Mueller–Hinton media (Ahman et al., 2020; Bilinskaya et al., 2020; Asempa et al., 2022). We gradually increased the zinc concentration of the test media starting with 0.0682 mM zinc (Baeza et al., 2019): Zinc-supplemented SAT revealed ceftazidime-avibactam resistance in eight of nine previously susceptible strains (Table 3). For a single strain (*E. coli* no. 700.18, NDM-5), elevated zinc in the SAT medium did not improve the detection of resistance. Previously, BMD of this *E. coli* (strain no. 700.18) had reliably determined resistance without supplemental zinc, suggesting that standard zinc levels should meet the individual zinc needs of this strain. This implies that for this strain sufficient zinc levels must coincide with additional traits provided by BMD, but not by the SAT system. These could be, for example, generally longer incubation times in BMD compared to the SAT system (18–24 h and 7.67–15.97 h, respectively) and/or the higher volume per test well (100 μL and 50 μL, respectively). Zinc-supplemented BMD revealed phenotypic ceftazidime-avibactam resistance in five of six previously susceptible strains (Table 3). Zinc-supplemented BMD of *P. mirabilis* strain no. 52.15 (VIM-1) was not clearly supportive for the detection of ceftazidime-avibactam resistance (with detection rates ≤ 50%). But SAT with addition of 0.1706 mM zinc had previously given consistently resistant MICs. Therefore, the SAT medium, but not the BMD medium, could include further, yet unknown components, which enhance the ceftazidime-avibactam AST of *P. mirabilis* no. 52.15, when combined with elevated zinc levels. BMD was performed again using BMD and SAT medium in parallel. *P. mirabilis* no. 52.15 was reliably categorized as resistant in BMD using zinc-supplemented SAT medium with 0.1706 or 0.2247 mM zinc (Table 3). Thus, in addition to zinc there are possibly further, critical, but yet unknown AST media components, which are relevant for ceftazidime-avibactam AST at least of individual MBL-harboring MDR-GN.

Zinc requirements of the individual ceftazidime-avibactam susceptible MBL-producing strains were not completely uniform, but ranged between 0.0682 mM and 0.1706 mM. Moreover, in SAT of three isolates, zinc supplementation was needed for the detection of ceftazidime-avibactam resistance, but not in BMD. This again supports the idea that there is no universally applicable minimum zinc concentration for AST of MBL-producing MDR-GN, but it depends on the individual strain and the test method.

In our study, zinc was added to test whether zinc content had an effect on the detection of ceftazidime-avibactam resistance in the two phenotypic AST methods used. However, modification of commercial AST media for routine diagnostic purposes is off label and not permissible, because it is not validated by the manufacturer and can impair the AST of other antimicrobial agents. This has been described for AST of *P. aeruginosa* and aminoglycosides, which is impaired when the content of divalent cations (Ca^2+^, Mg^2+^) is too high (CLSI, 2019). Nevertheless, our experimental setup demonstrated with two phenotypic AST platforms, that there are MDR-GN with functional MBLs that phenotypically exhibit ceftazidime-avibactam susceptibility when tested at standard zinc levels, but resistance when tested at elevated zinc levels. Furthermore, we demonstrated that also the strains that showed phenotypic resistance in AST with standard zinc content are subjected to the availability of zinc. After zinc depletion with the chelating agent EDTA, MICs of ceftazidime-avibactam and meropenem were distinctly reduced, which is consistent with decreased MBL activity (Supplementary Table 5).

There are further studies, whose findings match ours. Asempa et al. (2020) reported MBL-producing Enterobacterales with high meropenem MICs (>8 mg/L) in BMD with standard zinc levels (0.0147 ± 0.0006 mM). However, after zinc depletion of the test medium (<0.00003 mM), BMD could not detect phenotypic meropenem resistance of these MBL producers anymore. Next, the authors showed that in lungs of mice infected with MBL producers zinc was not detectable and meropenem treatment was effective in reducing the bacterial load. This suggests that due to low zinc bioavailability the hydrolytic activity of MBLs *in vivo* is significantly impaired. Decrease of meropenem, cefepime, and piperacillin-tazobactam MICs in BMD with zinc-depleted test medium could be confirmed in two follow-up studies (Bilinskaya et al., 2020; Abdelraouf et al., 2021). Furthermore, there are animal infection model studies that report *in vivo* activity of various β-lactam agents (including carbapenems and ceftazidime-avibactam) against MBL-harboring Enterobacterales with *in vitro* resistance provided that optimized dosing regimens were applied [reviewed in: Asempa et al., 2021a]. Given these findings, the hypothesis has arisen, that phenotypic resistance toward broad-spectrum β-lactams in MBL-producing isolates might be an *in vitro* artifact that depends on supraphysiological zinc concentrations in AST media and is not significant *in vivo* (Asempa et al., 2020). However, there are only anecdotal reports of human infections with MBL-harboring MDR-GN that were successfully treated with carbapenems (Chibabhai et al., 2018). Moreover, *in vitro* studies have shown that other metal ions can replace zinc in MBLs (for example BcII and VIM-2; Aitha et al., 2014; Cahill et al., 2016). MBLs with metal ions other than zinc can be able to hydrolyze meropenem, as demonstrated for Fe(II)-substituted BcII and VIM-2 (Cahill et al., 2016). This raises the possibility that other metal ions might allow for relevant hydrolytic activity of MBLs in human infections in the absence of zinc. Although a recent study reported lower meropenem MICs in AST with urine as test medium compared to AST with CAMHB (Asempa et al., 2021b), another investigation showed that meropenem AST with urine resulted in resistant MICs *in vitro*, suggesting that MBL-mediated, clinical resistance in urinary tract infections is possible (Hobson et al., 2020). Finally, additional host and pathogen factors that regulate zinc bioavailability and have not yet been explored are conceivable and could play a significant role for treatment success (Asempa et al., 2021a). Taken together, the clinical significance of low MBL activity and phenotypic susceptibility in zinc-deprived environments for human infections cannot be reliably assessed, because the available data are limited. Further studies are needed to address this issue and to test if the *in vitro* testing conditions correctly represent the situation *in vivo* (Bahr et al., 2021). Meanwhile, monotherapy of infections caused by MBL-harboring MDR-GN with broad-spectrum β-lactam antibiotics like meropenem or ceftazidime-avibactam is currently not encouraged (Rodriguez-Bano et al., 2018).

Overall, our findings show, that phenotypic ceftazidime-avibactam AST of MBL-producing MDR-GN can be challenging. A semi-automated AST system and BMD categorized nine and six MBL producers, respectively, as ceftazidime-avibactam susceptible, although production of functional MBLs was confirmed with the eCIM. Therefore, if carbapenem-resistant MDR-GN show phenotypical ceftazidime-avibactam susceptibility and treatment with this agent is considered, we recommend to exclude presence of MBLs with additional assays that specifically detect MBLs, for example, real-time PCR or immunochromatographic tests (Baeza et al., 2019; Gill et al., 2020a).

## Data availability statement

Sequence data are available online through the National Center for Biotechnology Information (NCBI) Sequence Read Archive (SRA) under BioProjects PRJNA869506 and PRJNA871076. The genome accession numbers are given in Supplementary Table 2.

## Author contributions

MS and JJ prepared the conceptualization of the study and wrote the original draft of the manuscript. MS, AHi, RGG, AS, and TR performed the experiments. MS, YP, RGG, AHa, and JJ developed the methodology of the study. Data analysis and presentation was done by MS and RGG. Resources were provided by YP, SGG, NP, and AG. All authors reviewed and edited the manuscript. All authors contributed to the article and approved the submitted version.

## Funding

This project was funded by the Bavarian Ministry of Science and the Arts in the framework of the Bavarian Research Network “New Strategies Against Multi-Resistant Pathogens by Means of Digital Networking—bayresq.net“, grant reference number: Kap. 1528 TG 83, given to AG.

## Conflict of interest

MS has received speaker fees from Becton Dickinson outside the submitted work. NP has received speaker or consultancy fees from bioMérieux, Pfizer, and Shionogi outside the submitted work. SGG has received speaker or consultancy fees from bioMérieux and Beckman Coulter not related to the submitted work. AHa has received speaker fees from bioMérieux and Beckman Coulter outside of the submitted work.

The remaining authors declare that the research was conducted in the absence of any commercial or financial relationships that could be construed as a potential conflict of interest.

## Publisher’s note

All claims expressed in this article are solely those of the authors and do not necessarily represent those of their affiliated organizations, or those of the publisher, the editors and the reviewers. Any product that may be evaluated in this article, or claim that may be made by its manufacturer, is not guaranteed or endorsed by the publisher.

## Footnotes

^1^ https://sourceforge.net/projects/bbmap/

^2^ https://github.com/tseemann/abricate
