# Supplementary material for: Increased zinc levels facilitate phenotypic detection of ceftazidime-avibactam resistance in metallo-β-lactamase-producing Gram-negative bacteria
Source: Front Microbiol. 2022 Nov 22;13:977330. doi: 10.3389/fmicb.2022.977330 (PMC9723239; doi:10.3389/fmicb.2022.977330)
Supplement: Supplementary file 1 [file Table_1.docx]

**Supplementary Table 1**

Primer sequences used for PCR amplification of different metallo-β-lactamase genes

| Primer name | Resistance genes | Sequence 5’---3’ | Product length | Reference |
| --- | --- | --- | --- | --- |
| NDM FWD | *bla*_NDM-type_ | CTGAGCACCGCATTAGCC | 754 bp | (Pfeifer et al., 2011) |
| NDM REV |  | GGGCCGTATGAGTGATTGC |  |  |
| VIM u FWD | *bla*_VIM-1/-2-type_ | AGTGGTGAGTATCCGACAG | 261 bp | (Grobner et al., 2009) |
| VIM u REV |  | ATGAAAGTGCGTGGAGAC |  |  |
| VIM-1 FWD | *bla*_VIM-1-type_ | TTATGGAGCAGCAACGATGT | 800 bp variable | This study |
| VIM-1 REV |  | AGCGATTTTTGTGTGCTTTG |  |  |
| VIM-1 FWD | *bla*_VIM-2-type_ | ATGTTCAAACTTTTGAGTAAG | 801 bp | This study |
| VIM-2 REV |  | CTACTCAACGACTGAGCG |  |  |
| IMP-1 FWD | *bla*_IMP-1-type_ | ATGAGCAAGTTATYWGTATTC | 762 bp | This study |
| IMP-1 REV |  | GCTGCAACGACTTGTTAG |  |  |
| IMP-2 FWD | *bla*_IMP-2-type_ | TCGAGAAGCTTGAAGAAGGTG | 615 bp | This study |
| IMP-2 REV |  | CAGCCTGTTCCCATGTACG |  |  |
| IMP-7 FWD | *bla*_IMP-7-type_ | TTGTAGCATTGCTGCCTCAG | 697 bp | This study |
| IMP-7 REV |  | TGGTTTTGATAGCTTTTTACTTTCG |  |  |

NDM, New Delhi Metallo-β-lactamase; VIM, Verona Integron Metallo-β-lactamase; IMP, Imipenemase Metallo-β-lactamase; *bla*, β-lactamase;

**References**

Grobner, S., Linke, D., Schutz, W., Fladerer, C., Madlung, J., Autenrieth, I.B., et al. (2009). Emergence of carbapenem-non-susceptible extended-spectrum beta-lactamase-producing Klebsiella pneumoniae isolates at the university hospital of Tubingen, Germany. J Med Microbiol 58(Pt 7), 912-922. doi: 10.1099/jmm.0.005850-0.

Pfeifer, Y., Wilharm, G., Zander, E., Wichelhaus, T.A., Gottig, S., Hunfeld, K.P., et al. (2011). Molecular characterization of blaNDM-1 in an Acinetobacter baumannii strain isolated in Germany in 2007. J Antimicrob Chemother 66(9), 1998-2001. doi: 10.1093/jac/dkr256.
