# Supplementary material for: Increased zinc levels facilitate phenotypic detection of ceftazidime-avibactam resistance in metallo-β-lactamase-producing Gram-negative bacteria
Source: Front Microbiol. 2022 Nov 22;13:977330. doi: 10.3389/fmicb.2022.977330 (PMC9723239; doi:10.3389/fmicb.2022.977330)
Supplement: Supplementary file 2 [file Table_2.docx]

**Supplementary Table 2**

Number of contigs and genome accessions of sequenced isolates

| Strain no. | Organism | # contigs | Genome accession^*^ |
| --- | --- | --- | --- |
| KR029 | *E. hormaechei* subsp. *hoffmannii* | 167 | JANUWP000000000 |
| 2.10 | *E. coli* | 22 | JANQAV000000000 |
| 371.12 | *E. coli* | 36 | JANQAY000000000 |
| 700.18 | *E. coli* | 11 | JANQAZ000000000 |
| KR016 | *E. coli* | 256 | JANUWO000000000 |
| KR089 | *E. coli* | 70 | JANUWN000000000 |
| KR140 | *E. coli* | 232 | JANUWM000000000 |
| KR148 | *E. coli* | 234 | JANUWL000000000 |
| WE3 | *E. coli* | 318 | JANUWK000000000 |
| 64.08 | *M. morganii* | 10 | JANQAX000000000 |
| 52.15 | *P. mirabilis* | 28 | JANQAW000000000 |
| 613.16 | *P. aeruginosa* | 81 | JANQBA000000000 |
| AV4 | *P. aeruginosa* | 99 | JANUXE000000000 |
| AV5 | *P. aeruginosa* | 183 | JANUXD000000000 |
| AV6 | *P. aeruginosa* | 134 | JANUXC000000000 |
| AV7 | *P. aeruginosa* | 138 | JANUXB000000000 |
| AV8 | *P. aeruginosa* | 111 | JANUXA000000000 |
| AV9 | *P. aeruginosa* | 143 | JANUWZ000000000 |
| AV10 | *P. aeruginosa* | 159 | JANUWY000000000 |
| AV11 | *P. aeruginosa* | 106 | JANUWX000000000 |
| AV12 | *P. aeruginosa* | 184 | JANUWW000000000 |
| AV13 | *P. aeruginosa* | 337 | JANUWV000000000 |
| AV15 | *P. aeruginosa* | 127 | JANUWU000000000 |
| KR112 | *P. aeruginosa* | 141 | JANUWT000000000 |
| KR119 | *P. aeruginosa* | 98 | JANUWS000000000 |
| WE5 | *P. aeruginosa* | 112 | JANUWR000000000 |
| WE10 | *P. aeruginosa* | 131 | JANUWQ000000000 |

***Data available at NCBI nucleotides: https://www.ncbi.nlm.nih.gov/nucleotide/**
