# Supplementary material for: Increased zinc levels facilitate phenotypic detection of ceftazidime-avibactam resistance in metallo-β-lactamase-producing Gram-negative bacteria
Source: Front Microbiol. 2022 Nov 22;13:977330. doi: 10.3389/fmicb.2022.977330 (PMC9723239; doi:10.3389/fmicb.2022.977330)
Supplement: Supplementary file 3 [file Table_3.docx]

**Supplementary Table 3**

Carbapenem MICs of 176 MBL-harboring Enterobacterales and *P. aeruginosa* isolates determined with the semi-automated antimicrobial susceptibility testing system

| Strain no. | Organism | MBL | Meropenem | | Imipenem | | Ertapenem | |
| --- | --- | --- | --- | --- | --- | --- | --- | --- |
|  |  |  | MIC (mg/L) | category | MIC (mg/L) | category | MIC (mg/L) | category |
| 81.16 | *C. freundii* complex | VIM-1 | 0.5 | S | 2 | S | >1 | R |
| 82.16 | *C. freundii* complex | VIM-1 | 8 | I | 8 | R | >1 | R |
| 77.16 | *C. freundii* complex | VIM-1 | 4 | I | 8 | R | >1 | R |
| 806.18 | *C. freundii* complex | VIM-1 | 8 | I | 8 | R | >1 | R |
| 733.14 | *E. cloacae* complex | VIM-4 | 8 | I | 8 | R | >1 | R |
| 52.17 | *E. cloacae* complex | NDM-1 | 8 | I | 8 | R | >1 | R |
| 437.17 | *E. cloacae* complex | NDM-1 | 8 | I | 4 | I | >1 | R |
| 319.19 | *E. cloacae* complex | NDM-1 | >8 | R | >8 | R | >1 | R |
| 129.08 | *E. cloacae* complex | VIM-1 | >8 | R | >8 | R | >1 | R |
| 144.09 | *E. cloacae* complex | VIM-1 | >8 | R | >8 | R | >1 | R |
| 151.09 | *E. cloacae* complex | VIM-1 | >8 | R | >8 | R | >1 | R |
| 201.09 | *E. cloacae* complex | VIM-1 | >8 | R | >8 | R | >1 | R |
| 220.09 | *E. cloacae* complex | VIM-1 | 8 | I | >8 | R | >1 | R |
| 92.09 | *E. cloacae* complex | VIM-1 | >8 | R | >8 | R | >1 | R |
| 125.09 | *E. cloacae* complex | VIM-1 | 8 | I | 8 | R | >1 | R |
| 32.11 | *E. cloacae* complex | VIM-1 | >8 | R | >8 | R | >1 | R |
| 16.11 | *E. cloacae* complex | VIM-1 | >8 | R | >8 | R | >1 | R |
| 550.12 | *E. cloacae* complex | VIM-1 | 8 | I | 8 | R | >1 | R |
| 637.15 | *E. cloacae* complex | VIM-1 | >8 | R | >8 | R | >1 | R |
| 585.15 | *E. cloacae* complex | VIM-1 | 8 | I | >8 | R | >1 | R |
| 348.15 | *E. cloacae* complex | VIM-1 | 4 | I | 4 | I | >1 | R |
| 136.15 | *E. cloacae* complex | VIM-1 | >8 | R | >8 | R | >1 | R |
| 506.15 | *E. cloacae* complex | VIM-1 | 8 | I | 8 | R | >1 | R |
| 34.16 | *E. cloacae* complex | VIM-1 | 8 | I | 8 | R | >1 | R |
| 8.17 | *E. cloacae* complex | VIM-1 | >8 | R | >8 | R | >1 | R |
| 485.17 | *E. cloacae* complex | VIM-1 | >8 | R | >8 | R | >1 | R |
| 533.17 | *E. cloacae* complex | VIM-1 | >8 | R | >8 | R | >1 | R |
| 516.17 | *E. cloacae* complex | VIM-1 | 8 | I | >8 | R | >1 | R |
| 555.17 | *E. cloacae* complex | VIM-1 | 8 | I | >8 | R | >1 | R |
| 48.18 | *E. cloacae* complex | VIM-1 | 8 | I | 8 | R | >1 | R |
| 665.18 | *E. cloacae* complex | VIM-1 | >8 | R | >8 | R | >1 | R |
| 837.18 | *E. cloacae* complex | VIM-1 | 8 | I | >8 | R | >1 | R |
| 469.19 | *E. cloacae* complex | VIM-1 | 8 | I | 8 | R | >1 | R |
| 108.19 | *E. cloacae* complex | VIM-1 | 2 | S | 4 | I | 1 | R |
| 143.19 | *E. cloacae* complex | VIM-1 | >8 | R | >8 | R | >1 | R |
| 407.19 | *E. cloacae* complex | VIM-1 | >8 | R | >8 | R | >1 | R |
| 417.19 | *E. cloacae* complex | VIM-1 | >8 | R | >8 | R | >1 | R |
| 602.18 | *E. cloacae* complex | VIM-1 | 8 | I | 8 | R | >1 | R |
| 1.20 | *E. cloacae* complex | VIM-1 | >8 | R | >8 | R | >1 | R |
| AV2 | *E. cloacae* complex | VIM-1 | 4 | I | 8 | R | >1 | R |
| AV3 | *E. cloacae* complex | NDM-1 | >8 | R | >8 | R | >1 | R |
| KR29 | *E. cloacae* complex | VIM-1 | 0.5 | S | 8 | R | 0.5 | S |
| 2.10 | *E. coli* | NDM-1 | >8 | R | >8 | R | >1 | R |
| 371.12 | *E. coli* | NDM-1 | >8 | R | >8 | R | >1 | R |
| 69.15 | *E. coli* | NDM-1 | >8 | R | >8 | R | >1 | R |
| 699.15 | *E. coli* | NDM-1 | >8 | R | >8 | R | >1 | R |
| 416.17 | *E. coli* | NDM-1 | >8 | R | >8 | R | >1 | R |
| 700.18 | *E. coli* | NDM-5 | >8 | R | >8 | R | >1 | R |
| 159.19 | *E. coli* | NDM-5 | >8 | R | >8 | R | >1 | R |
| 161.19 | *E. coli* | NDM-1 | >8 | R | 8 | R | >1 | R |
| 663.18 | *E. coli* | VIM-1 | 8 | I | 8 | R | >1 | R |
| 162.10 | *E. coli* | VIM-1 | >8 | R | >8 | R | >1 | R |
| KR89 | *E. coli* | NDM-1 | >8 | R | >8 | R | >1 | R |
| KR148 | *E. coli* | NDM-1 | 4 | I | 4 | I | >1 | R |
| KR16 | *E. coli* | NDM-1 | 8 | I | 8 | R | >1 | R |
| KR140 | *E. coli* | NDM-5 | >8 | R | >8 | R | >1 | R |
| WE3 | *E. coli* | NDM-5 | >8 | R | >8 | R | >1 | R |
| 200.11 | *K. oxytoca* complex | VIM-2 | >8 | R | >8 | R | >1 | R |
| 52.07 | *K. oxytoca* complex | VIM-1 | >8 | R | >8 | R | >1 | R |
| 142.07 | *K. oxytoca* complex | VIM-1 | >8 | R | >8 | R | >1 | R |
| 98.08 | *K. oxytoca* complex | VIM-1 | 4 | I | 8 | R | 1 | R |
| 91.09 | *K. oxytoca* complex | VIM-1 | >8 | R | 8 | R | >1 | R |
| 370.12 | *K. oxytoca* complex | VIM-1 | 4 | I | 8 | R | 1 | R |
| 91.13 | *K. oxytoca* complex | VIM-1 | 8 | I | 8 | R | >1 | R |
| 543.13 | *K. oxytoca* complex | VIM-1 | >8 | R | >8 | R | >1 | R |
| 594.15 | *K. oxytoca* complex | VIM-1 | >8 | R | >8 | R | >1 | R |
| 783.15 | *K. oxytoca* complex | VIM-1 | >8 | R | >8 | R | >1 | R |
| 226.17 | *K. oxytoca* complex | VIM-1 | >8 | R | >8 | R | >1 | R |
| KR59 | *K. oxytoca* complex | VIM-type | 1 | S | 8 | R | >1 | R |
| KR52 | *K. oxytoca* complex | VIM-type | 2 | S | >8 | R | 1 | R |
| 93.10 | *K. pneumoniae* | NDM-1 | >8 | R | >8 | R | >1 | R |
| 39.11 | *K. pneumoniae* | NDM-1 | >8 | R | >8 | R | >1 | R |
| 50.11 | *K. pneumoniae* | NDM-1 | >8 | R | >8 | R | >1 | R |
| 596.13 | *K. pneumoniae* | NDM-1 | 4 | I | 8 | R | >1 | R |
| 384.13 | *K. pneumoniae* | NDM-1 | >8 | R | >8 | R | >1 | R |
| 633.13 | *K. pneumoniae* | NDM-1 | >8 | R | >8 | R | >1 | R |
| 1123.14 | *K. pneumoniae* | NDM-1 | >8 | R | >8 | R | >1 | R |
| 1122.14 | *K. pneumoniae* | NDM-1 | >8 | R | >8 | R | >1 | R |
| 166.14 | *K. pneumoniae* | NDM-1 | >8 | R | >8 | R | >1 | R |
| 431.14 | *K. pneumoniae* | NDM-1 | >8 | R | >8 | R | >1 | R |
| 872.14 | *K. pneumoniae* | NDM-1 | >8 | R | >8 | R | >1 | R |
| 334.15 | *K. pneumoniae* | NDM-1 | >8 | R | >8 | R | >1 | R |
| 336.15 | *K. pneumoniae* | NDM-1 | >8 | R | >8 | R | >1 | R |
| 340.15 | *K. pneumoniae* | NDM-1 | >8 | R | >8 | R | >1 | R |
| 339.15 | *K. pneumoniae* | NDM-1 | >8 | R | >8 | R | >1 | R |
| 338.15 | *K. pneumoniae* | NDM-1 | >8 | R | >8 | R | >1 | R |
| 589.15 | *K. pneumoniae* | NDM-1 | >8 | R | >8 | R | >1 | R |
| 164.16 | *K. pneumoniae* | NDM-1 | >8 | R | >8 | R | >1 | R |
| 1057.16 | *K. pneumoniae* | NDM-1 | >8 | R | >8 | R | >1 | R |
| 53.17 | *K. pneumoniae* | NDM-1 | >8 | R | >8 | R | >1 | R |
| 75.17 | *K. pneumoniae* | NDM-1 | >8 | R | >8 | R | >1 | R |
| 76.17 | *K. pneumoniae* | NDM-1 | >8 | R | >8 | R | >1 | R |
| 479.17 | *K. pneumoniae* | NDM-1 | >8 | R | >8 | R | >1 | R |
| 435.17 | *K. pneumoniae* | NDM-1 | >8 | R | >8 | R | >1 | R |
| 875.18 | *K. pneumoniae* | NDM-1 | >8 | R | >8 | R | >1 | R |
| 684.18 | *K. pneumoniae* | NDM-1 | >8 | R | >8 | R | >1 | R |
| 326.18 | *K. pneumoniae* | NDM-1 | >8 | R | 8 | R | >1 | R |
| 245.19 | *K. pneumoniae* | NDM-1 | >8 | R | >8 | R | >1 | R |
| 115.19 | *K. pneumoniae* | NDM-1 | 4 | I | 4 | I | >1 | R |
| 147.19 | *K. pneumoniae* | NDM-1 | >8 | R | >8 | R | >1 | R |
| 162.19 | *K. pneumoniae* | NDM-1 | >8 | R | 8 | R | >1 | R |
| 300.14 | *K. pneumoniae* | VIM-19 | 8 | I | >8 | R | >1 | R |
| 187.10 | *K. pneumoniae* | VIM-4 | >8 | R | 8 | R | >1 | R |
| 404.08 | *K. pneumoniae* | VIM-4 | 8 | I | >8 | R | >1 | R |
| 62.07 | *K. pneumoniae* | VIM-1 | >8 | R | >8 | R | >1 | R |
| 5.08 | *K. pneumoniae* | VIM-1 | >8 | R | >8 | R | >1 | R |
| 152.09 | *K. pneumoniae* | VIM-1 | >8 | R | >8 | R | >1 | R |
| 115.09 | *K. pneumoniae* | VIM-1 | >8 | R | >8 | R | >1 | R |
| 156.09 | *K. pneumoniae* | VIM-1 | >8 | R | >8 | R | >1 | R |
| 219.09 | *K. pneumoniae* | VIM-1 | >8 | R | >8 | R | >1 | R |
| 163.10 | *K. pneumoniae* | VIM-1 | >8 | R | >8 | R | >1 | R |
| 164.10 | *K. pneumoniae* | VIM-1 | >8 | R | >8 | R | >1 | R |
| 49.11 | *K. pneumoniae* | VIM-1 | >8 | R | >8 | R | >1 | R |
| 188.11 | *K. pneumoniae* | VIM-1 | >8 | R | >8 | R | >1 | R |
| 556.12-1 | *K. pneumoniae* | VIM-1 | >8 | R | >8 | R | >1 | R |
| 269.19 | *K. pneumoniae* | VIM-1 | >8 | R | 8 | R | >1 | R |
| AV1 | *K. pneumoniae* | NDM-type | >8 | R | >8 | R | >1 | R |
| KR202 | *K. pneumoniae* | NDM-type | >8 | R | >8 | R | >1 | R |
| KR88 | *K. pneumoniae* | NDM-type | >8 | R | >8 | R | >1 | R |
| KR21 | *K. pneumoniae* | NDM-1 | 8 | I | >8 | R | >1 | R |
| KR44 | *K. pneumoniae* | NDM-type | >8 | R | >8 | R | >1 | R |
| KR85 | *K. pneumoniae* | NDM-type | >8 | R | >8 | R | >1 | R |
| KR45 | *K. pneumoniae* | NDM-type | >8 | R | >8 | R | >1 | R |
| KR43 | *K. pneumoniae* | NDM-type | >8 | R | >8 | R | >1 | R |
| KR198 | *K. pneumoniae* | NDM-type | >8 | R | >8 | R | >1 | R |
| KR199 | *K. pneumoniae* | NDM-type | >8 | R | >8 | R | >1 | R |
| KR79 | *K. pneumoniae* | VIM-1 | >8 | R | >8 | R | >1 | R |
| 64.08 | *M. morganii* | VIM-1 | 2 | S | >8 | R | 1 | R |
| 110.19 | *P. aeruginosa* | VIM-2 | >8 | R | >8 | R | nd | - |
| 6.18 | *P. aeruginosa* | VIM-2 | >8 | R | >8 | R | nd | - |
| 52.18-1 | *P. aeruginosa* | VIM-2 | >8 | R | >8 | R | nd | - |
| 1005.14 | *P. aeruginosa* | VIM-2 | >8 | R | >8 | R | nd | - |
| 376.14 | *P. aeruginosa* | VIM-2 | >8 | R | >8 | R | nd | - |
| 747.17 | *P. aeruginosa* | VIM-2 | >8 | R | >8 | R | nd | - |
| 613.16 | *P. aeruginosa* | VIM-2 | >8 | R | >8 | R | nd | - |
| 617.16 | *P. aeruginosa* | VIM-2 | >8 | R | >8 | R | nd | - |
| 64.15-2 | *P. aeruginosa* | VIM-2 | >8 | R | >8 | R | nd | - |
| 27.14 | *P. aeruginosa* | VIM-2 | >8 | R | >8 | R | nd | - |
| 156.14 | *P. aeruginosa* | VIM-2 | >8 | R | >8 | R | nd | - |
| 33.14 | *P. aeruginosa* | VIM-2 | >8 | R | >8 | R | nd | - |
| 239.13 | *P. aeruginosa* | VIM-2 | >8 | R | >8 | R | nd | - |
| 347.12 | *P. aeruginosa* | VIM-2 | >8 | R | >8 | R | nd | - |
| 504.15 | *P. aeruginosa* | VIM-2 | 4 | I | >8 | R | nd | - |
| 584.15 | *P. aeruginosa* | VIM-2 | >8 | R | >8 | R | nd | - |
| 468.12 | *P. aeruginosa* | VIM-2 | >8 | R | >8 | R | nd | - |
| 470.12 | *P. aeruginosa* | VIM-2 | 4 | I | >8 | R | nd | - |
| 359.11 | *P. aeruginosa* | VIM-2 | >8 | R | >8 | R | nd | - |
| 360.11 | *P. aeruginosa* | VIM-2 | >8 | R | >8 | R | nd | - |
| 369.11 | *P. aeruginosa* | VIM-6 | >8 | R | >8 | R | nd | - |
| 47.09 | *P. aeruginosa* | VIM-28 | >8 | R | >8 | R | nd | - |
| 230.10 | *P. aeruginosa* | VIM-type | >8 | R | >8 | R | nd | - |
| 40.14 | *P. aeruginosa* | IMP-7 | >8 | R | >8 | R | nd | - |
| 82.10 | *P. aeruginosa* | IMP-1 | >8 | R | >8 | R | nd | - |
| 140.12 | *P. aeruginosa* | VIM-2 | >8 | R | >8 | R | nd | - |
| 484.12 | *P. aeruginosa* | VIM-1 | >8 | R | >8 | R | nd | - |
| AV4 | *P. aeruginosa* | VIM-2 | >8 | R | >8 | R | nd | - |
| AV5 | *P. aeruginosa* | VIM-2 | >8 | R | >8 | R | nd | - |
| AV6 | *P. aeruginosa* | VIM-2 | >8 | R | >8 | R | nd | - |
| AV7 | *P. aeruginosa* | VIM-2 | >8 | R | >8 | R | nd | - |
| AV8 | *P. aeruginosa* | VIM-2 | >8 | R | >8 | R | nd | - |
| AV9 | *P. aeruginosa* | VIM-2 | >8 | R | >8 | R | nd | - |
| AV10 | *P. aeruginosa* | VIM-2 | >8 | R | >8 | R | nd | - |
| AV11 | *P. aeruginosa* | VIM-1 | >8 | R | >8 | R | nd | - |
| AV12 | *P. aeruginosa* | VIM-2 | >8 | R | >8 | R | nd | - |
| AV13 | *P. aeruginosa* | VIM-2 | >8 | R | >8 | R | nd | - |
| WE5 | *P. aeruginosa* | VIM-2 | >8 | R | >8 | R | nd | - |
| WE10 | *P. aeruginosa* | VIM-2 | >8 | R | >8 | R | nd | - |
| KR112 | *P. aeruginosa* | IMP-7 | >8 | R | >8 | R | nd | - |
| KR119 | *P. aeruginosa* | VIM-2 | >8 | R | >8 | R | nd | - |
| AV15 | *P. aeruginosa* | VIM-2 | >8 | R | >8 | R | nd | - |
| 164.19 | *P. mirabilis* | NDM-1 | 8 | I | nd | - | >1 | R |
| 215.17 | *P. mirabilis* | NDM-1 | 8 | I | nd | - | >1 | R |
| 795.15 | *P. mirabilis* | NDM-1 | 8 | I | nd | - | >1 | R |
| 52.15 | *P. mirabilis* | VIM-1 | >8 | R | nd | - | ≤0.25 | S |
| 505.15 | *P. stuartii* | VIM-1 | >8 | R | >8 | R | >1 | R |
| 15.11 | *S. marcescens* | VIM-1 | >8 | R | >8 | R | >1 | R |

MBL, metallo-β-lactamase; MIC, minimal inhibitory concentration; IMP, Imipenemase Metallo-β-lactamase; NDM, New Delhi Metallo-β-lactamase; VIM, Verona Integron Metallo-β-lactamase; S, susceptible; I, susceptible, dose-dependent; R, resistant; nd, not done;
