# Supplementary material for: Increased zinc levels facilitate phenotypic detection of ceftazidime-avibactam resistance in metallo-β-lactamase-producing Gram-negative bacteria
Source: Front Microbiol. 2022 Nov 22;13:977330. doi: 10.3389/fmicb.2022.977330 (PMC9723239; doi:10.3389/fmicb.2022.977330)
Supplement: Supplementary file 4 [file Table_4.docx]

**Supplementary Table 4**

Quantitative bacterial culture of NDM-5-harboring *E. coli* strain no. 700.18 with and without zinc addition to the SAT medium.

| Zinc addition (mM) | w/o | 0.0682 | 0.1137 | 0.1365 | 0.1706 | 0.2274 | *P* value^a^ |
| --- | --- | --- | --- | --- | --- | --- | --- |
| CFU/mL x 10^8^ (mean ± SD) | 6.1 ± 0.5 | 7.1 ± 1.2 | 6.9 ± 0.4 | 6.0 ± 0.8 | 6.7 ± 0.8 | 6.0 ± 0.6 | 0.311 |
| Range (CFU/mL x 10^8^) | 5.6-6.4 | 5.9-8.2 | 6.5-7.2 | 5.2-6.8 | 6.2-7.6 | 5.3-6.3 |  |

NDM, New Delhi Metallo-β-lactamase; SAT, semi-automated testing; CFU, colony forming units; w/o, without; SD, standard deviation;

^a^ Kruskall-Wallis test
