# Supplementary material for: Increased zinc levels facilitate phenotypic detection of ceftazidime-avibactam resistance in metallo-β-lactamase-producing Gram-negative bacteria
Source: Front Microbiol. 2022 Nov 22;13:977330. doi: 10.3389/fmicb.2022.977330 (PMC9723239; doi:10.3389/fmicb.2022.977330)
Supplement: Supplementary file 5 [file Table_5.docx]

**Supplementary Table 5**

Phenotypic ceftazidime-avibactam antimicrobial susceptibility testing of four MBL-producing MDR-GN in un-modified and zinc-depleted test media. EDTA was used for zinc depletion.

|  |  |  |  |  | Semi-automated AST system | |  | Broth microdilution | |
| --- | --- | --- | --- | --- | --- | --- | --- | --- | --- |
| Strain no. | Organism | EDTA (mg/L) | MBL | β-lactam | EDTA | |  | EDTA | |
|  |  |  |  |  | without | with |  | without | with |
| 162.10 | *E. coli* | 300 | VIM-1 | Ceftazidime-avibactam | R | S |  | R | S |
|  |  |  |  | Meropenem | R | S |  | S-I^1^ | S^2^ |
| 39.11 | *K. pneumoniae* | 300 | NDM-1 | Ceftazidime-avibactam | R | S |  | R | S |
|  |  |  |  | Meropenem | R | S |  | R | S |
| 82.10 | *P. aeruginosa* | 150 | IMP-1 | Ceftazidime-avibactam | R | S |  | R | S |
|  |  |  |  | Meropenem | R | S |  | R^3^ | S-I^4^ |
| 52.18-1 | *P. aeruginosa* | 50 | VIM-2 | Ceftazidime-avibactam | R | S |  | R | S |
|  |  |  |  | Meropenem | R | S |  | R^5^ | I^6^ |

MBL, metallo-β-lactamase; MDR-GN, multidrug-resistant Gram-negative bacteria; AST, antimicrobial susceptibility testing; MIC, minimal inhibitory concentration; VIM, Verona Integron Metallo-β-lactamase; NDM, New Delhi Metallo-β-lactamase; IMP, Imipenemase Metallo-β-lactamase;

Semi-automated AST and BMD were performed in parallel in four test runs.

^1^ Meropenem MIC (mg/L): 2/4/2/4

^2^ Meropenem MIC (mg/L): ≤0.125/≤0.125/≤0.125/≤0.125

^3^ Meropenem MIC (mg/L): >16/>16/>16/>16

^4^ Meropenem MIC (mg/L): 4/2/1/2

^5^ Meropenem MIC (mg/L): >16/>16/>16/>16

^6^ Meropenem MIC (mg/L): 4/4/4/4
